# Implementation and evaluation of the human papillomavirus (HPV) vaccination pilot for men who have sex with men (MSM), England, April 2016 to March 2017

**DOI:** 10.2807/1560-7917.ES.2019.24.8.1800055

**Published:** 2019-02-21

**Authors:** Michael Edelstein, Nalini Iyanger, Nicola Hennessy, David Mesher, Marta Checchi, Kate Soldan, Mark McCall, Julie Nugent, Jonathan Crofts, Janice Lo, Richard Gilson, Karen Powell, Mary Ramsay, Joanne Yarwood

**Affiliations:** 1 Immunisation and countermeasures division, National Infection Service, Public Health England, London, United Kingdom; 2 Blood Safety, Hepatitis, Sexually Transmitted Infections (STI) and HIV service, National Infection Service, Public Health England, London, United Kingdom; 3 Mortimer Market Sexual Health Clinic, London, United Kingdom

**Keywords:** human papillomavirus, HPV, vaccine, vaccination, England, men who have sex with men, MSM, sexual health, policy, sexually transmitted infections, immunisation

## Abstract

Background: Opportunistic human papillomavirus (HPV) vaccination for men who have sex with men (MSM) was piloted in sexual health clinics (SHC) in England between 2016 and 2018.

Aim: to evaluate the pilot’s first year (April 2016–March 2017) in terms of feasibility, acceptability, uptake, impact and equity and interpret the outcome in the context of wide HPV vaccination policy.

Methods: Attendance and uptake data from routine SHC surveillance datasets and a cross-sectional survey administered to individuals receiving the vaccine were analysed.

Results: Among 18,875 eligible MSM, 8,580 (45.5%) were recorded as having received one HPV vaccine dose, decreasing slightly with increasing age, and uptake was higher in rural than urban areas. Survey results suggested that of those receiving the first dose of HPV vaccine, 8% were new attendees and that among those, less than 11% attended just to receive the vaccine. Of those having their first HPV vaccination, 95% indicated they would like to receive the next vaccine doses at the same clinic and 85% of patients reported accessing other services when visiting SHC for the first dose of vaccine.

Conclusion: An opportunistic HPV vaccination programme for MSM can be delivered in an acceptable and, as far as can be evaluated, equitable manner, without major disruption to SHC and HIV clinics.

## Introduction

Infection with human papillomavirus (HPV) is associated with 99% of cervical cancers [1] and almost all genital warts (GW) [2]. HPV is also associated with some vulval, vaginal, penile, anal and head and neck cancers [3-5]. In September 2008, vaccination against HPV was introduced into the school-based vaccination programme for girls aged 12–13 years in the United Kingdom (UK), with the primary aim to protect girls from cervical cancer. The programme initially offered a bivalent vaccine that protects against HPV infection with high-risk HPV types 16 and 18, responsible for around 80% of cervical cancers in the UK [6]. From 2012, the quadrivalent HPV (qHPV) vaccine (Gardasil) was offered according to the recommended three-dose schedule administered at 0, 2 and 6 months. In addition to HPV types 16 and 18, The qHPV vaccine protects against HPV infection with the low-risk HPV types 6 and 11, responsible for around 90% of GW [2]. A female only HPV vaccination programme is expected to provide indirect herd protection to males when vaccination coverage is high [7].

Men who have sex with men (MSM) receive very little indirect benefit from a vaccination programme that only targets girls [8]. In Australia, 5 years after the introduction of the HPV vaccine in girls, GW incidence had decreased in females and heterosexual males aged less than 30 years, but not in MSM [9]. Furthermore, compared with heterosexual males, MSM have higher rates of HPV infection and HPV-related disease including GW [10] and anal cancer [11]. In July 2018, the government announced that boys aged 12–13 years will also be offered the HPV vaccine as part of the school-based vaccination programme in the UK [12]. However, fully protecting MSM through a teenage gender-neutral HPV vaccination programme will take decades.

In November 2015, the Joint Committee on Vaccination and Immunisation (JCVI) advised that a three-dose HPV vaccination programme targeting MSM aged up to 45 years, who attend sexual health clinics (SHC)/HIV clinics, should be undertaken subject to procurement of the vaccine and delivery of the programme at a cost-effective price [13]. The advice was based on an analysis carried out to determine the impact and cost-effectiveness of HPV vaccination of MSM in England [8].

To ascertain the complexities associated with the commissioning and delivery of a HPV vaccination programme involving SHC/HIV clinics in England, Public Health England (PHE) together with its partners set up an HPV immunisation pilot for MSM (HPV MSM). The objectives of the pilot were to evaluate the acceptability, feasibility, equity, vaccine uptake and impact of such a programme on services delivered through SHC/HIV clinics in order to inform a potential future national programme. The outcome of the pilot were summarised in a PHE report [14]. This paper describes the implementation of the HPV MSM pilot in England in 2016-17, disseminates more details regarding the methods and outcomes of the pilot study to a wider audience and discusses relevance of an HPV MSM programme in the context of existing female and gender-neutral HPV vaccination programmes. To our knowledge, this pilot was the first nationally planned and funded HPV vaccination programme specifically targeting MSM. It is hoped that the lessons outlined here may also be relevant to other countries considering what HPV vaccination strategy to adopt for MSM.

## Methods

### Data sources

Genitourinary medicine clinic activity dataset (GUMCAD) is a mandatory reporting system providing data on sexual health services (SHS) and sexually transmitted disease (STI) diagnoses from all commissioned SHS in England [15]. This surveillance data are collected by SHC and submitted to PHE.

HIV and AIDS Reporting System (HARS) collects data on patients diagnosed with HIV infection attending all National Health Service (NHS) HIV outpatient services in England [16]. In both data sources, MSM who attend can be identified.

### Setting and pilot sites selection

In England, it is estimated that 3.1% of the male population was gay or bisexual in 2014, although among those 18–44 years this proportion ranged from 4.2–4.7% [17] or ca 475,000 individuals [18]. In the same year, around 140,000/475,000 MSM (29.5% of MSM) attended SHC clinics at least once [19].

Implementation of the pilot in SHC/HIV clinics, based on recommendations from JCVI, assumed that MSM attending these clinics are more like to participate in high-risk sexual behaviour and have a higher number of sexual partners than other MSM and that these factors would contribute to making the programme cost effective [8]. In addition, unlike in general practice, MSM can be systematically identified in SHC/HIV settings as sexual risk is recorded.

To evaluate the pilot in different settings e.g. urban/rural and high/low MSM prevalence areas, PHE selected clinics that were geographically spread across England. SHC/HIV clinics were classified as ‘Urban major conurbation’, ‘Urban city and town’, or ‘Rural village and dispersed’, based on the Lower Super Output Area (LSOA, a geographical area of between 1,000 and 3,000 individuals [20]) of the clinic. Based on these criteria, selected SHC/HIV clinics were contacted and asked to express an interest in participating in the pilot.

Information materials for patients and healthcare professionals e.g. a patient information leaflet, training and clinical guidelines were developed and made available online [21].

### Human papillomavirus vaccination

MSM eligible for vaccination were defined as MSM up to the age of 45 years, attending SHC clinics who were not previously vaccinated with HPV vaccine. MSM meeting the inclusion criteria were opportunistically offered three doses regardless of sexual risk or disease status. The number of doses was based on national recommendations as at 2016 for individuals aged 15 and over [22] only a few individuals under 15 years attend SHC/HIV clinics.

On average, around half of MSM who attend a SHC return at least twice in the following 24-month period [19]; to facilitate the delivery of three doses as part of existing clinic appointments the timeframe for delivery was extended to 24 months. This schedule was deemed clinically acceptable providing there was a gap of at least 1 month between the first two doses and 3 months between the second and third doses, in line with national guidance [22].

The number of vaccines allocated to each region was based on the number of MSM attenders in the previous year extracted from the GUMCAD STI Surveillance System [15] and HARS [16]. For allocation purposes, we ensured there were sufficient doses for an equivalent of 100% uptake for dose one, 80% for dose two and 70% for dose three. Each region was responsible for vaccine allocation to individual clinics, within the allocated limit. Clinics were asked to ensure that sufficient vaccine was kept for second and third doses.

Vaccine could be ordered free of charge through ImmForm, an online platform for ordering and stock management of centrally procured vaccine. Clinics could claim a £10 (€11.30) per dose administration fee from PHE. Clinics were asked to report any vaccine wasted e.g. by accident/cold chain failure/expiry before use and were requested to hold no more than a 2- week supply of HPV vaccine and instead opt to reorder as required to limit stockpiling and potential vaccine wastage.

To ensure the HPV vaccine doses used in the pilot could be monitored independently from the teenage female programme, efforts were made to ensure that the ordering of HPV vaccine for the pilot study remained separate from the school-based HPV vaccination programme for girls. Some initial errors were identified, but these were promptly resolved; it was expected and accepted, however, that a certain level of cross use between the two programmes would occur.

### Evaluation

### Patient survey

A patient questionnaire was designed and administered to evaluate the feasibility, acceptability, equity and impact of a potential opportunistic HPV vaccination programme for MSM on SHC/HIV clinics. Questions included: the reason for attendance to the clinic; whether or not they were first time attenders; if they had heard about the pilot and where; why they had chosen the particular clinic they were attending; and their opinion on the most suitable setting for receiving the vaccine and subsequent doses.

The SHC/HIV clinics were grouped in 29 clusters based on what SHS the clinics belonged to. For each cluster, the number of questionnaires required, based on an estimated number of eligible attendees in each clinic, was calculated to have a 95% confidence interval (95% CI) of a maximum of +/− 5% around each question at the cluster level. Overall, a minimum of 7,109 responses were required to attain the required precision in each cluster. Each clinic was required to administer the questionnaire to consecutive individuals receiving the vaccine until the sample size was reached. Answers were weighted in order to adjust for different response rates in different clusters. Questionnaires where the clinic could not be identified were discarded. The proportion of responses to each question was reported overall, together with 95% CI.

In order to maximise response rate, MSM receiving the vaccine were asked to fill the paper-based questionnaire immediately after vaccination while still in the clinic.

#### GUMCAD/HARS

Feasibility and acceptability of the HPV MSM pilot were evaluated by measuring first qHPV dose uptake among eligible MSM, calculated cumulatively as the proportion of eligible MSM attending SHC clinics offering the vaccine who had received the first dose. The number of eligible MSM increased over the study period as more clinics started offering the HPV vaccine. Data from eight clinics were excluded from all uptake analyses due to under recording of HPV vaccination administration as a result of incomplete use of new codes.

Equity was also assessed by comparing uptake in urban major conurbations, urban city and towns and rural villages and dispersed populations, and between different age groups.

GUMCAD data was also used to evaluate the impact on clinic attendance by comparing participating clinics in the pilot with those that did not participate.

This evaluation was considered as a routine service evaluation and formal ethical approval was therefore not required.

## Results

Between April 2016 and March 2017, 42 of 43 invited SHC/HIV clinics from seven of nine English regions took part in the pilot HPV MSM study. This represented ca 20% of all level three SHC clinics (specialist clinics providing complex services) covering around a third of the approximately 140,000 eligible MSM who attended that year; Among the clinics included in the analysis, 18,875 eligible MSM attended clinics during the time the vaccine was available. The median age among MSM was 31 years (interquartile range: 26–38 years). Overall, 8,580 (45.5%) were recorded as having received the first dose. First dose uptake decreased slightly with increasing age, from 51.2% in MSM aged 25 years and under to 36.9% in MSM aged 41–45 years. Compared with urban areas, uptake of HPV vaccination in rural areas was higher (Table 1).

**Table 1:** Uptake of first human papillomavirus vaccine dose and survey responses, by clinic setting, England, April 2016–March 2017

Setting	First dose qHPV vaccine uptake		Aware of recommendation for MSM to receive vaccine % (95% CI)	Next dose at same clinic % (95% CI)	Next dose at GP % (95% CI)	Accessed other services as part of attendance % (95% CI)
	n/N	%				
Major urban conurbation	6,243/13,773	45.3	31.3 (30.0–32.5)	92.7 (92.1–93.3)	6.3 (5.7–6.9)	85.4 (84.4–86.3)
Urban city and town	1,919/4,330	44.3	31.5 (29.4–33.7)	92.7 (91.3–93.9)	10.5 (9.1–12.1)	85.7 (84.1–87.2)
Rural village and dispersed	418/772	54.1	23.3 (0.2–26.9)	89.2 (86.29–91.51)	12.2 (9.7–15.3)	82.7 (79.3–85.6)

CI: confidence interval; GP: general practice; MSM: men who have sex with men; qHPV: quadrivalent human papillomavirus.

Source: Public Health England [14].

Uptake of the first HPV vaccine dose among all attending MSM did not begin to increase substantially until October 2016 (Figure), reflecting the start of HPV MSM pilot implementation at the majority of participating clinics. Only two of the total 42 clinics had implemented HPV vaccination for MSM in June 2016 and further clinics did not implement the HPV MSM pilot until September 2016. Prior to the start of the pilot, between January 2009 and May 2016, overall attendance in all SHC clinics increased by 4.8% a year on average [15]. Pilot clinic MSM attendances increased from 12,762 in June 2016 (at the start of the pilot) to 13,382 in March 2017 (4.5% increase). During the same time period, non-pilot clinics attendances did not change substantially (11,444–11,705).

**Figure:**
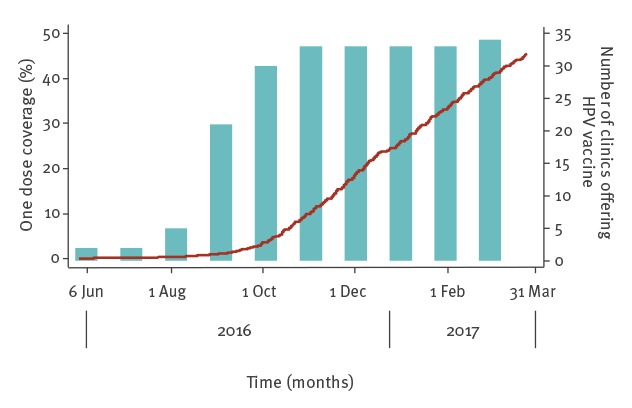
Human papillomavirus vaccine first dose uptake among eligible men who have sex with men in pilot clinics, England, April 2016–March 2017 (n = 8,580)

HPV: human papillomavirus; SHC: sexual health centre.

Source: Adapted from Public Health England [14].

### Survey results

Overall, 9,009 questionnaire responses from 25/29 clusters were received of which 8,554 were used in the evaluation analysis. As the eligibility to answer specific questions was dependant on responses to previous questions, the number of responses to individual questions ranged quite widely (range: 8,554–4,137). The proportions presented here are weighted so may differ from crude proportions obtained by dividing the number of responses to specific questions by the number of responders. Both crude and weighted proportions are presented in Table 2.

**Table 2:** Survey answers from men who have sex with men receiving first dose of human papillomavirus vaccine, England, April 2016–March 2017 (n = 8,554)

Question		Responders (n)	Response	Responses (n)	Crude %	Weighted %^a^	95% CI
Have you ever attended a SHC/HIV clinic before?		8,511	Yes	7,734	90.9	91.8	91.3–92.4
			No	777	9.1	8.1	7.6–9.2
Is this your usual/local clinic?		8,499	Yes	7,293	85.8	86.8	86–87.5
			No	1,206	14.2	13	12.5–14.0
Did you know before attending the clinic that the HPV vaccination is recommended for MSM?		8,503	Yes	2,709	31.9	31.1	30–32.2
			No	5,794	68.1	68.9	67.8–70
Was getting the HPV vaccine the main reason that you attended the clinic today?^b^	Overall	4,137	Yes	512	12.4	12.4	11.3–13.6
			Yes, but also had another reason	521	12.6	11.1	10.1–12.2
			No	3,104	75	76.5	75–77.9
	New attendees to SHC/HIV	366	Yes	38	10.4	10.8	7.7–14.8
			Yes, but also had another reason	47	12.8	11.2	8.5–14.6
			No	281	76.8	78	73.5–82
	Previously attended SHC/HIV	3,758	Yes	469	12.5	12.4	11.2–13.7
			Yes, but also had another reason	470	12.5	11	10–12.2
			No	2,819	75	76.6	75–78.1
Did you access any other services at the clinic?		8,469	Yes	7,169	84.6	85.4	84.5–86.1
			No	1,300	15.4	14.6	13.9–15.5
Why did you choose to attend this particular clinic?		8,486	Clinic I usually attend	5,313	62.6	66.1	65.1–67.1
			My local clinic	2,018	23.8	19.8	19.1–20.6
			Most convenient for me	2,226	26.2	25.1	24.1–26.1
			I wanted vaccine and my local clinic does not have it	115	1.4	1.6	0.9–1.4
			I wanted vaccine and knew this clinic provided it	205	2.4	2.2	1.9–2.6
			Other reason	744	8.8	9.0	8.3–9.7
Where would you like to have your next vaccine dose?		8,368	At this clinic	7,821	93.5	94.6	94.2–95.2
			Clinic closer to where I live	500	6	5	4.6–5.5
			Clinic closer to where I work	241	2.9	2.2	1.9–2.6
			High street pharmacy	339	4.1	4.1	3.6–4.6
			My GP practice	677	8.1	7.2	6.6–7.7
			Other	6	0.1	0.05	0.002–0.2

CI: confidence interval; GP: general practice; HIV: human immunodeficiency virus; HPV: human papillomavirus; MSM: men who have sex with men; SHC: sexual health clinic.

^a ^Proportions are weighted, so may differ from dividing the number of responses to specific questions by the number of responders.

^b ^Only those who answered ‘yes’ to question three were eligible to answer.

Source: Adapted from Public Health England [14].

Of MSM that responded to the questionnaire, 91.8% (95% CI 91.3–92.4) had previously attended a SHC/HIV clinic and 85.4% (95% CI 84.5–86.1) had accessed other services as part of their attendance for the HPV vaccine (Table 2). Overall, 12.4% (95% CI: 11.3–13.6) of responders attended specifically to receive the vaccine. Among new attendees this proportion was 10.8% (95% CI: 7.7–14.8).

In response to the question regarding where they would like to receive the next HPV vaccination doses, 94.6% (95% CI: 94.1.6–95.1) indicated they would like to receive the next doses at the same clinic. General practice was mentioned by 7.2% (95% CI: 6.6–7.7) as a potential setting for the next HPV vaccine dose. Among those attending clinics in rural areas, this proportion increased to 12.2% (95% CI: 9.7–15.3) although SHC remained the preferred setting (Table 1)

## Discussion

To our knowledge, the English HPV MSM vaccination pilot is the first to specifically target MSM as part of a centrally planned and implemented process. An MSM programme is relevant to countries with an adolescent HPV vaccination programme regardless of whether it is female only or gender neutral. HPV vaccine uptake data and survey results suggest it is feasible to deliver HPV vaccination opportunistically to MSM through SHC/HIV clinics.

All countries in the European Union and European Economic Area (EU/EEA) include HPV as part of their routine vaccination schedule [23] either as a female only programme or as a gender neutral one. England has had a female only programme and the decision to implement a gender neutral HPV programme was announced in July 2018; an initiative that has already been taken by other countries in Europe, North America and Oceania [24]. The UK programme will include vaccinating both boys and girls aged 12–13 years (school year 8) against HPV, who will remain eligible for vaccination until the age of 18 years in case they are not vaccinated at school. However, it will take decades until all MSM up to the age of 45 will have been vaccinated through the national school-based programme. In the meantime, unvaccinated MSM outside the routine adolescent cohort will remain at higher risk for HPV-related diseases and the targeted HPV MSM programme will be needed to protect this high-risk group.

### Implementation of a HPV MSM programme

Implementing an MSM programme presents some operational challenges including the identification of eligible individuals, the choice of an appropriate setting, delivering the programme in a cost effective manner and monitoring vaccine ordering and uptake. In England, sexual orientation is not routinely recorded in primary care medical records and it is, therefore, difficult to initiate vaccine courses in primary care i.e. the setting where most vaccine programmes are administered. However, a model where a vaccination programme is initiated in SHC/HIV clinics in England, with subsequent doses delivered at the GP could be envisaged. Nevertheless, without a setting where MSM can routinely be identified, such a programme is difficult to monitor and countries envisaging to initiate a HPV vaccination programme for MSM should consider such monitoring issues ahead of implementation.

Results from the evaluation survey suggested that SHC/HIV clinics were the preferred setting in which to receive the HPV vaccine. The pilot study had a led to a very modest number of new MSM who had specifically attended the SHC/HIV clinics to receive the vaccine; indicating that the additional workload to deliver a HPV vaccination programme (in addition to other services provided in clinics) would be acceptable. The majority of MSM who received the HPV vaccine at SHC/HIV clinics were existing patients attending for other reasons, who were vaccinated opportunistically. Attendance data from GUMCAD is consistent with these findings, with no change in overall trends in attendance to SHC and a modest increase in attendance in pilot clinics compared with others. Uptake of HPV vaccine was higher in rural clinics, but lower in MSM aged 41 and higher. These findings warrant further investigation.

The introduction of the HPV vaccination pilot for MSM has been positive and SHC/HIV clinics, Lesbian, Gay, Bisexual and Transgender (LGBT) and sexual health charities have been supportive.

In England the cost effectiveness threshold for public health intervention is £20–30,000 (€23-34,000)/QALY [25]; the expected reduction in GW incidence is major contributor to the cost-effectiveness of the HPV vaccine in MSM [8]. The MSM programme benefits from a procurement price that reflects the large volume of HPV vaccine ordered to cover an annual cohort of 350,000 girls. Any vaccine changes to the school-based HPV programme may therefore have cost implications for the MSM HPV programme. Thus, implementation of a cost-effective programme may be challenging in countries without an existing school-based programme, in countries with a programme using the bivalent HPV vaccine (i.e. not including HPV types 6 and 11 against GW) or in countries with no centralised vaccine procurement.

### Limitations

Uptake figures are likely underestimated in this pilot study, with more HPV vaccine doses being administered than being recorded, due to a number of known data recording issues. Although the clinics where recording was particularly poor were not included in uptake estimates, the under recording issues also affected other clinics. Anecdotal discussions with several clinics highlighted that a proportion of patients with no vaccination recorded were in fact vaccinated. Additionally, data were only available for the first 10 months of the HPV MSM pilot, and it is not unexpected therefore that coverage would be lower in these early stages. Despite the potential for underestimation, the uptake during the pilot study was promising and would indicate that a HPV MSM programme would be acceptable to the target population.

Using a paper-based questionnaire presents some drawbacks, in particular over costs and inconvenience of postal returns. The high number of returns relative to the target for each clinic suggested high levels of interest and engagement from patients and service providers. Questionnaires were anonymised and undated, so it was not possible to calculate a questionnaire response rate but clinics reported that very few individuals refused to fill the questionnaire.

### Conclusions

The UK was one of the first countries to introduce a HPV vaccine programme specifically targeting MSM. In order to answer specific questions with regards to feasibility, equity, vaccine uptake and impact on services, a pilot approach was taken in England. The pilot concluded in 2017/18 and based on its outcome a national programme began being rolled out in April 2018. The evaluation of the English pilot suggests that an opportunistic HPV vaccination programme for MSM is best delivered in SHC/HIV clinics and can be delivered equitably and with minimal disruption to those services. A HPV MSM vaccination programme alongside a high-coverage teenage vaccination programme confers protection to most individuals at risk. Even in countries with a gender-neutral programme, an MSM programme will protect individuals at high risk of HPV infection for the decades it will take for all eligible MSM to have been offered vaccination as teenagers. Surveillance activities to monitor the impact of an MSM programme on HPV infection, genital warts and HPV-related cancers will be conducted. These studies are needed to confirm the cost-effectiveness of HPV vaccination for MSM.
